# Identification of Neuromuscular Performance Parameters as Risk Factors of Non-contact Injuries in Male Elite Youth Soccer Players: A Preliminary Study on 62 Players With 25 Non-contact Injuries

**DOI:** 10.3389/fspor.2021.615330

**Published:** 2021-10-18

**Authors:** Mathias Kolodziej, Kevin Nolte, Marcus Schmidt, Tobias Alt, Thomas Jaitner

**Affiliations:** ^1^Department of Strength and Conditioning and Performance, Borussia Dortmund, Dortmund, Germany; ^2^Institute for Sports and Sport Science, Technical University (TU) Dortmund University, Dortmund, Germany; ^3^Department of Biomechanics, Performance Analysis and Strength and Conditioning, Olympic Training and Testing Centre Westphalia, Dortmund, Germany

**Keywords:** injury prevention, risk factors, neuromuscular, performance, youth elite soccer players, biomechanical screening

## Abstract

**Introduction:** Elite youth soccer players suffer increasing numbers of injuries owing to constantly increasing physical demands. Deficits in neuromuscular performance may increase the risk of injury. Injury risk factors need to be identified and practical cut-off scores defined. Therefore, the purpose of the study was to assess neuromuscular performance parameters within a laboratory-based injury risk screening, to investigate their association with the risk of non-contact lower extremity injuries in elite youth soccer players, and to provide practice-relevant cut-off scores.

**Methods:** Sixty-two elite youth soccer players (age: 17.2 ± 1.1 years) performed unilateral postural control exercises in different conditions, isokinetic tests of concentric and eccentric knee extension and knee flexion (60°/s), isometric tests of hip adduction and abduction, and isometric tests of trunk flexion, extension, lateral flexion and transversal rotation during the preseason period. Non-contact lower extremities injuries were documented throughout 10 months. Risk profiling was assessed using a multivariate approach utilizing a Decision Tree model [Classification and Regression Tree (CART) method].

**Results:** Twenty-five non-contact injuries were registered. The Decision Tree model selected the COP sway, the peak torque for knee flexion concentric, the functional knee ratio and the path of the platform in that hierarchical order as important neuromuscular performance parameters to discriminate between injured and non-injured players. The classification showed a sensitivity of 0.73 and a specificity of 0.91. The relative risk was calculated at 4.2, meaning that the risk of suffering an injury is four times greater for a player, who has been classified as injured by the Decision Tree model.

**Conclusion:** Measuring static postural control, postural control under unstable condition and the strength of the thigh seem to enable a good indication of injury risk in elite youth soccer players. However, this finding has to be taken with caution due to a small number of injury cases. Nonetheless, these preliminary results may have practical implications for future directions in injury risk screening and in planning and developing customized training programs to counteract intrinsic injury risk factors in elite youth soccer players.

## Introduction

Prospective research provide data on injury incidences of youth soccer players, especially from the amateur level (Timpka et al., 2007; Schmikli et al., 2011; Brito et al., 2012b). However, it has been shown that the injury incidences rises with the playing level (Pfirrmann et al., 2016). A growing database is available for elite youth soccer players to analyze injury incidences (Cloke et al., 2009, 2011; Moore et al., 2011). The overall injury incidence in elite youth soccer players varies between 2.0 and 19.4 injuries per 1,000 h of exposure (Le Gall et al., 2006; Deehan et al., 2007; Pfirrmann et al., 2016; Renshaw and Goodwin, 2016). Injury incidences increase linearly with age- especially from the age of 14 onwards- with 16- and 18-year-old players have an injury incidence similar to or higher than that of adult players (Peterson et al., 2000; Price et al., 2004; Pfirrmann et al., 2016). The majority of all injuries in youth soccer players (70–88%) occur to the lower extremities, affecting the knee and ankle joints, as well as the thigh/hip muscles (Price et al., 2004; Pfirrmann et al., 2016). Up to 72% of lower extremity injuries in elite youth soccer players are reportedly non-contact injuries (Price et al., 2004; Renshaw and Goodwin, 2016).

Altered neuromuscular performance is indicated as the underlying mechanisms of non-contact lower extremity injuries that occur during soccer-specific movements, such as running, cutting, landing and side-stepping (Price et al., 2004; Read et al., 2016). “Neuromuscular” describes the complex interaction between sensory, motor and central integration and processing components involved in maintaining functional joint stability (Riemann and Lephart, 2002). Therefore, neuromuscular performance can be considered as the ability of the neuromuscular system to functionally control and drive movements by an appropriate use and coordination of muscular strength and endurance, muscle recruitment pattern, proprioceptive feedback, and reflex activity (Faude et al., 2017). Deficits in neuromuscular performance may potentially increase the risk of injury and neuromuscular injury prevention programs, including postural control and strength exercises, have been shown to reduce the risk of lower extremity injuries (Mandelbaum et al., 2005; Abernethy and Bleakley, 2007; Lehr et al., 2017). In detail, neuromuscular performance parameters such as the strength of the thigh, the hip and the trunk muscles, as well as postural control, have been suggested to be primary modifiable risk factors for lower extremity injuries in different populations (Tropp et al., 1984; Leetun et al., 2004; Read et al., 2015). However, there is a paucity of prospective studies on neuromuscular performance parameters as risk factors for lower extremity injuries in youth male elite soccer players.

Read et al. (2016) have proposed a hypothetical hierarchical injury risk factor model for knee and ankle ligament injuries in youth soccer players. It is a theoretical model and based on previously identified neuromuscular imbalances for adult females and males. In a first prospective examination of a few proposed risk factors derived from the theoretical model, Read et al. (2018) conducted a field-based screening battery. They identified peak landing vertical ground reaction force asymmetry during the single leg countermovement jump as the most prominent injury risk factor in under 11 to under 12 and under 15 to under 16 players. Within the age groups under 13 to under 14 and under 18s, no neuromuscular risk factors were significantly associated with risk of injury (Read et al., 2018). Rommers et al. (2020) assessed the injury risk in elite youth soccer using a machine learning approach. They identified a higher predicted age at peak height velocity, higher body height and leg length, lower fat percentage and average performance on the standing broad jump as the five most important predictors for injury.

In the available literature, the investigations by Read et al. (2018) and Rommers et al. (2020) are the only studies investigating prospective neuromuscular performance parameters to evaluate risk factors for lower extremity injuries in youth elite soccer players. However, the researchers used field-based screening tests such as the single leg hop for distance, 75% of maximum hop and stick, the standing broad jump, curl-ups and the Körperkoordinationstest für Kinder (KTK), whose measurement properties and relationship to injury are currently limited, conflicting or unknown (Hegedus et al., 2015). In addition, prospective studies to identify cut-off scores, which is the first step toward a validated screening program (Bahr, 2016), are missing.

Therefore, the purpose of the study was to assess neuromuscular performance parameters within a laboratory-based injury risk screening, to investigate their association with the risk of non-contact lower extremity injuries in elite youth soccer players, and to provide practice-relevant cut-off scores.

## Materials and Methods

### Study Design and Participants

This study was a prospective cohort study. Six teams (under 16, under 17, and under 19) of the youth academies of two professional German soccer clubs were contacted and invited to participate. Three teams (under 16, under 17, and under 19) agreed to take part in the study. All members of the playing squad for each team received written information on the purpose of the study, the preseason screening and the injury documentation. If the players were not of full age, the parents gave their consent. Immediately before testing, the players were asked to complete a questionnaire on paper to collect demographics, background information and the histories of any previous lower extremity injuries. A research assistant guided the interview. All lower extremity injuries up to 6 months prior to the assessments had to be reported. Players were excluded if they wore a prophylactic device (e.g., ankle brace), if they reported a recent (<6 weeks) musculoskeletal injury or if they had any physical complaints (e.g., a head injury) that might have impaired their performance. Players who met the inclusion criteria were tested via injury risk screening at the beginning of the preparation period of the 2018/2019 season. The Ethics Committee of TU Dortmund University confirmed that the requirements of the Declaration of Helsinki were met.

### Injury Data Collection

The data collection followed the consensus statement on injury definitions and data collection procedures in studies of soccer injuries (Fuller et al., 2006). Only time-loss injuries were counted, meaning that a player was missing completely in training or match play. Contact injuries, illnesses and injuries to the upper body were excluded because of their unpredictable nature compared to non-contact injury mechanisms. Injuries were documented by a standardized injury form throughout 10 months of the season 2018/2019 starting after the initial testing at the beginning of the preparation period. Physiotherapists of the respective team completed the injury forms and communicated them once a month to the researcher. Training and match hours were documented for each player by the coaching staff. Injury incidence was taken to be the number of injuries per 1,000 h of exposure time, calculated by dividing the total number of injuries by the total exposure time and multiplying the result by 1,000.

### Injury Risk Screening

#### Testing Protocol

**Figure 1:**
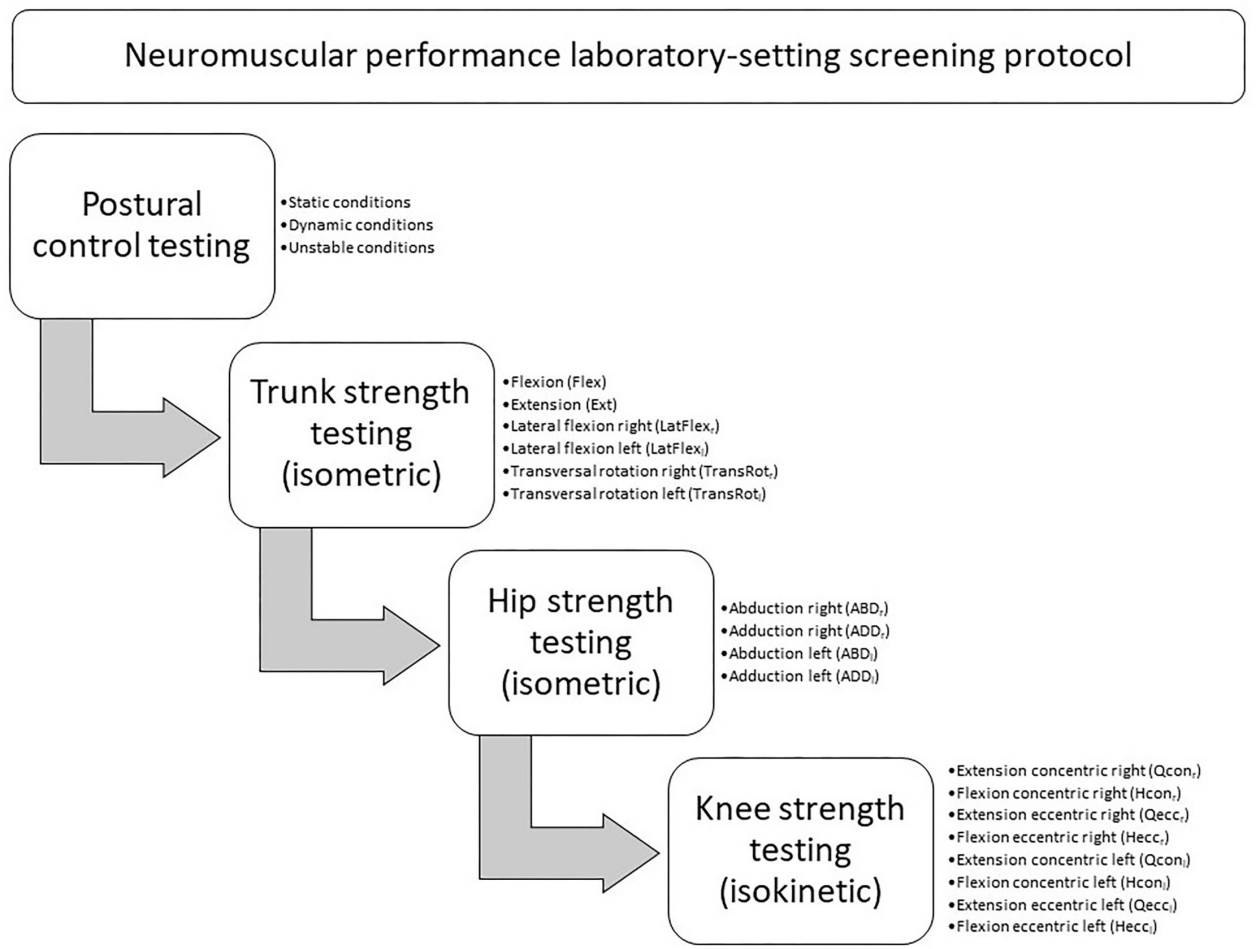
Neuromuscular performance laboratory-setting screening protocol.

Prior to testing, all players performed a standardized warm-up, including 5 min on a bike ergometer, followed by movement preparation and plyometric exercises. After the warm-up, the players underwent the tests in a standardized order to avoid neuromuscular fatigue in the lower extremities and the trunk, particularly while undergoing the postural control testing, and to ensure equal test conditions for all players (see Figure 1).

##### Postural Control Testing

Postural control was assessed by three unilateral exercises with different conditions to meet the dynamic nature of soccer activities (static postural control, dynamic postural control and postural control under unstable conditions). Furthermore, previous data have identified weak relationships between static and dynamic tasks used to assess postural control in youth male soccer players, thus different postural control tests are necessary (Pau et al., 2015; Ringhof and Stein, 2018).

First, static postural control was measured by a single-legged stance test on a force plate (AMTI, Inc., Watertown, MA, USA). The players were instructed to start on the right leg and to maintain balance for 10 s in a static position with the eyes open. The hands were placed on the hips and the swinging leg was flexed 90° in the hip, knee and ankle to minimize contributions from the contralateral leg (Frisch et al., 2011).

Dynamic postural control was analyzed using the Dynamic Postural Stability Index (DPSI) devised by Wikstrom et al. (2005). Dynamic postural control can be defined as the ability to maintain balance while transitioning from a dynamic to a static state (Goldie et al., 1989). The original jump protocol has been modified to simulate soccer-specific movement. The players started in a standing position 70 cm from the center of the force plate. They were asked to jump off with both legs and to perform an imaginary header before landing with one leg on the force plate. Each player was to land first on the right leg, stabilize as quickly as possible and balance for 3 s with hands on hips and looking straight ahead with the eyes open (Wikstrom et al., 2007).

Postural control under unstable conditions was measured while single-leg standing on a multi-axial free-swinging platform (Posturomed, Haider Bioswing, Pullenreuth, Germany). The quadratic support platform of the Posturomed guarantees free movement in the medial-lateral and anterior-posterior direction (Boeer et al., 2010). After familiarization with the test device, the players were instructed to start on the right leg and to maintain balance for 20 s in a static position with the eyes open. The hands were placed on the hips, and the swinging leg was flexed 90° in the hip, knee and ankle to minimize contributions from the contralateral leg (Frisch et al., 2011).

For all three testing conditions, the trial was repeated if the player lost balance and touched the floor with the contralateral leg or if he failed to maintain a unilateral stance, either by moving the stance foot from the starting position or by removing the hands from the hips. Three trials were performed on each leg for each testing condition.

##### Strength Testing

**Figure 2:**
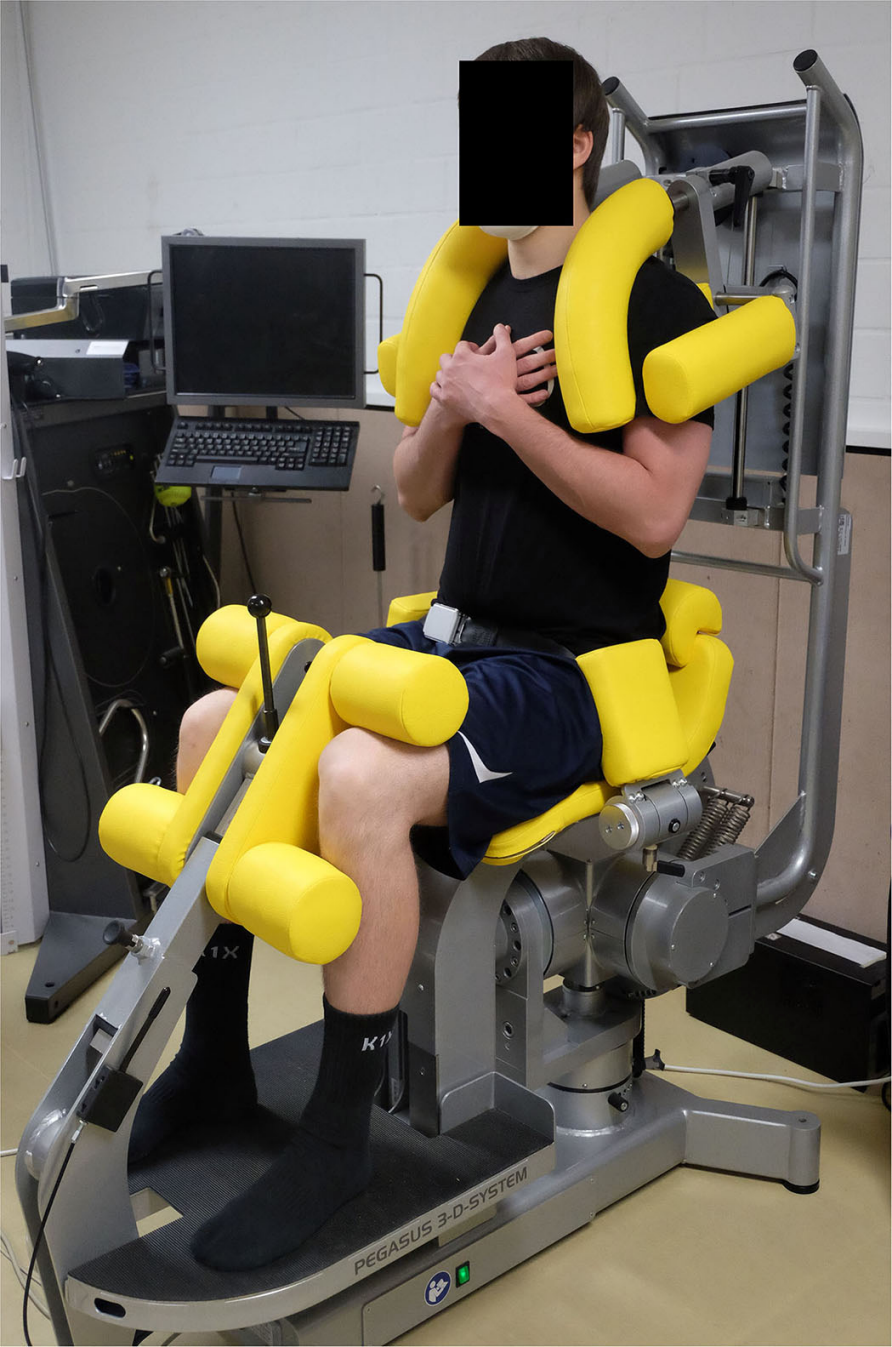
Set-up of the Pegasus 3-D system and participant positioning for the trunk strength testing.

Isometric trunk flexion, extension, lateral flexion and transversal rotation was measured in all anatomic movement planes (sagittal–frontal–transverse) with the Pegasus 3-D system (Biofeedback Motor Control GmbH, Leipzig, Germany). The players were seated in an upright position with the hips and knees flexed at an angle of 90° on the dynamometer chair. To avoid segmental body movement, adapters were attached to both knees, both hips and both shoulders (Kocjan and Sarabon, 2014). Furthermore, a belt was used to stabilize the pelvis (see Figure 2).

The players warmed up by making one submaximal isometric contraction (~70% maximum voluntary contraction, MVC) of 5 s prior to the following testing condition (Figure 1). After a 1-min rest, the players performed three maximal voluntary isometric contractions of 5 s with a 1-min rest period in between for each of the four testing directions (see Figure 1) (Meyer et al., 2013).

**Figure 3:**
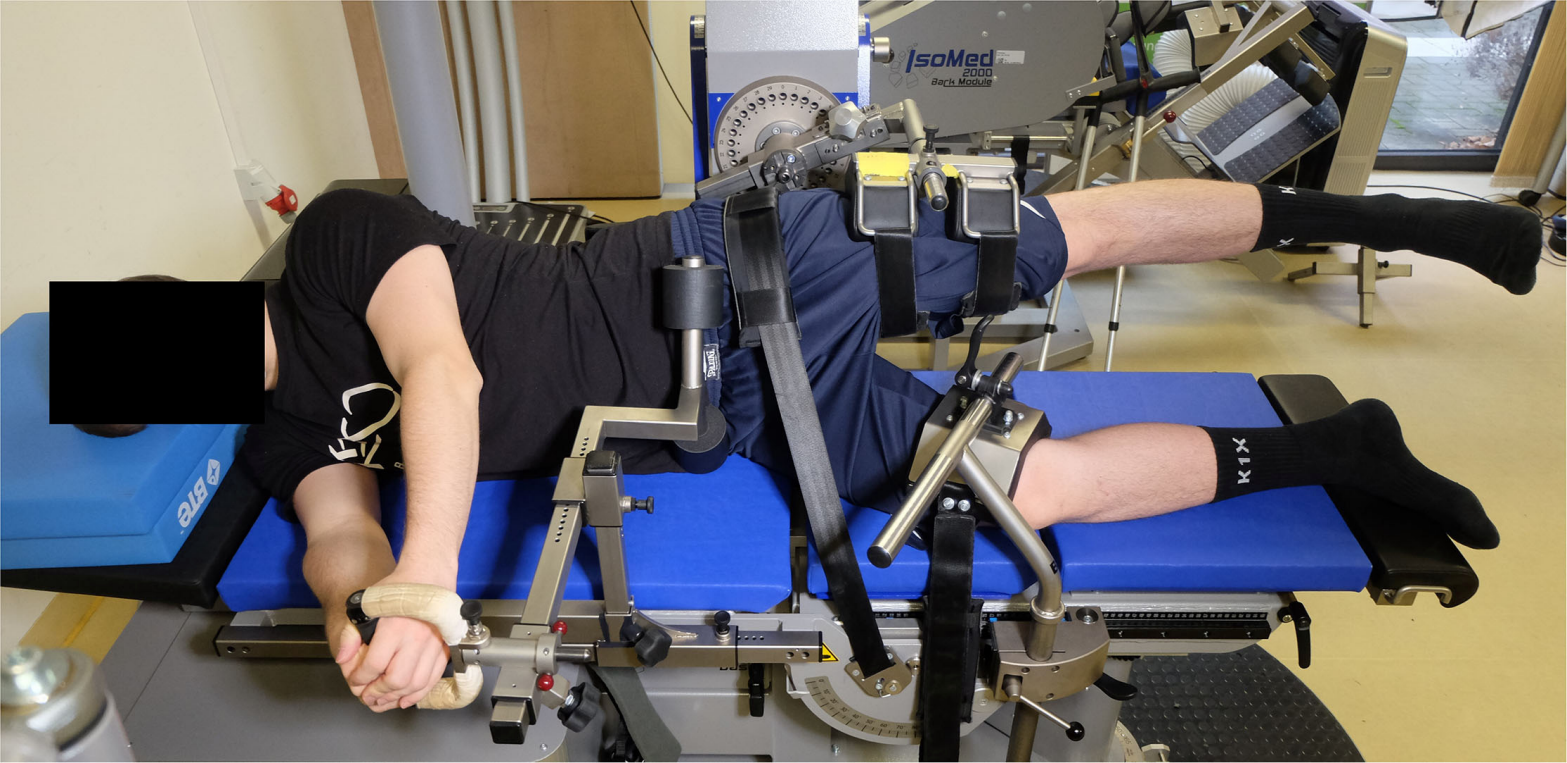
Set-up of the IsoMed 2000 dynamometer and participant positioning for the hip strength testing.

Isometric hip adduction and abduction was measured by an isokinetic dynamometer IsoMed 2000 (D&R Ferstl GmbH, Hemau, Germany). The players were attached on their sides to the dynamometer chair in a standardized position to limit segmental body movement (Meyer et al., 2013). They were asked to take off their shoes to minimize the effect of gravity on torque production. To minimize trunk and pelvic rotation during testing, adapters were attached at the lumbar spine and the ilium. The players were allowed to hold a grip in front of them to ensure that the back remained in a neutral position (Laheru et al., 2007). The tested leg was secured into the dynamometer pad 2 cm above the center of the knee joint and strapped at the femur to avoid hip rotation. The non-tested hip and knee were flexed slightly for comfort and stabilization and fixed to the dynamometer chair (Meyer et al., 2013). Finally, the dynamometer height, chair and dynamometer fore–aft distances were adjusted to ensure that the dynamometer pivot corresponded to the greater trochanter level. The starting position of the tested leg was set for the abduction at 0° and for the adduction at 10° (Maffiuletti, 2010) (see Figure 3).

A gravity correction measurement was conducted by the integrated software for each leg. The players warmed up by making one submaximal isometric contraction (~70% MVC) of 5 s prior to the following testing condition (Figure 1). After a 1-min rest, the players performed three maximal voluntary isometric contractions of 5 s with a 1-min rest period in between (Meyer et al., 2013).

**Figure 4:**
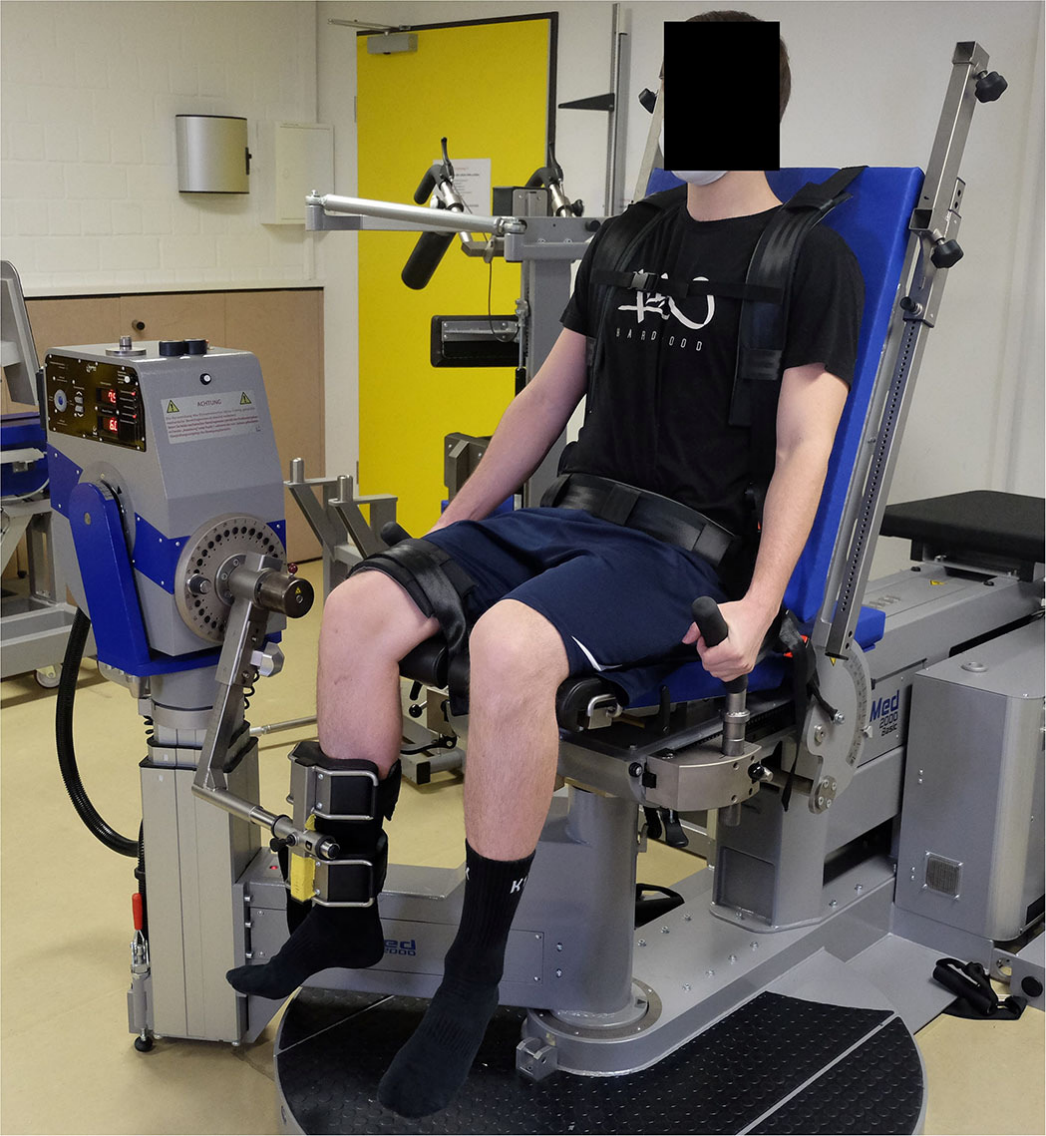
Set-up of the IsoMed 2000 dynamometer and participant positioning for the knee strength testing.

Finally, the measurement of concentric and eccentric knee extension and flexion was performed by an isokinetic dynamometer IsoMed 2000 (D&R Ferstl GmbH, Hemau, Germany). A shin pad for unilateral knee flexion and extension was attached to the motor-driven axis of the active dynamometer. The players were seated in an upright position on the dynamometer chair and secured with straps across the shoulder, chest and hip in a standardized position. The backrest was tilted at 75°. The range of motion was set at 0° (full extension) to 90° (flexion) (Fousekis et al., 2010). The mechanical axis of the dynamometer was aligned with the lateral femoral epicondyle of the player's knee. To minimize accelerative inaccuracies, the players were asked to take off their shoes. At a 90° flexed knee, the distal part of the shin pad of the dynamometers lever arm was fixed by a strap ~2–3 cm proximal to the lateral malleolus of the ankle (see Figure 4).

A gravity correction measurement was conducted by the integrated software for each leg. Adequate familiarization with the dynamometer was provided in the form of six submaximal repetitions (~50% MVC) at both testing conditions (extension and flexion) and both contraction modes (concentric and eccentric) at the testing speed of 60°/s (Hassani et al., 2006). Based on the testing order (Figure 1), the players performed two submaximal repetitions (70% of MVC) followed by three maximal repetitions at 60°/s, with a 1-min rest between each testing condition and each contraction mode (Van Dyk et al., 2016).

#### Data Processing and Outcome Measures

##### Postural Control Testing

For postural control under static and dynamic conditions, ground reaction force data were collected at a sampling frequency of 1,000 Hz. The raw force and moment signals arising from the foot–force–plate interface were filtered using a fourth-order low-pass filter with a cut-off frequency of 5 Hz (Knapp et al., 2011).

For postural control under the static condition, the COP was calculated from the force and moment signals using DASYLab data acquisition software (DASYLab, Datalog, Mönchengladbach, Germany). The COP sway (cm) was calculated for each leg and each trial from the COP data, using a custom R routine according to the following formula:


$$COP\;sway=\sum \sqrt{{\left(x_{i}-x_{i-1}\right)}^{2}+{\left(y_{i}-y_{i-1}\right)}^{2}}$$

The lowest COP sway for each leg was determined. The mean of both limbs was used for further analysis.

For postural control under the dynamic condition, the average magnitude of the ground reaction force vector around zero points in the medial–lateral direction (frontal plane), anterior–posterior direction (sagittal plane) and vertical direction (vertical plane) of the force plate was calculated. The medial-lateral stability index and anterior-posterior stability index assess the fluctuations from zero along the frontal and sagittal axes of the force plate, respectively. The vertical stability index assesses the fluctuation from the subject's body weight (Wikstrom et al., 2007). The DPSI is a composite of the stability indices in the three directions and is sensitive to changes in all three directions. The DPSI was calculated using a custom R routine according to the formula by Wikstrom et al. (2006):


$$\begin{array}{l} DPSI=\frac{\sqrt{\sum {\left(0-x\right)}^{2}+\sum {\left(0-y\right)}^{2}+\sum {\left(body\;weight-z\right)}^{2}}}{number\;of\;data\;points} \end{array}$$

The ground contact phase was defined as the ground reaction force of the foot strike on the force plate exceeding a threshold of 50 N of the smoothed vertical ground reaction force. Data were reduced to 3-s post-landing time frames. The lowest DPSI for each leg of three trials was determined. The mean of both limbs was used for further analysis.

For postural control under the unstable condition, the motions of the platform were recorded in two perpendicular directions (anterior-posterior and medial-lateral) and at a 50 Hz sample rate by a manufacturer's accelerometer, which was attached to the center of the platform's bottom. The manufacturer's software provided the raw data after converting the analog signals through a 14 bit A/D converter. According to the manufacturer's guidelines (MicroSwing 6 handbook, Haider Bioswing, Pullenreuth, Germany), the raw data of the accelerometer were integrated twice using a custom R routine, resulting in the total path of the platform (mm). The shortest path of the platform (mm) for each leg was determined. The mean of both limbs was used for statistical analysis.

##### Strength Testing

For trunk strength testing, raw data were recorded at a sampling rate of 100 Hz, using the manufacturer's software (BioMC, Biofeedback Motor Control GmbH, Leipzig, Germany). The highest isometric peak torque (Nm) for trunk flexion, trunk extension, trunk lateral flexion (right and left) and trunk transversal rotation (right and left) was determined. Each relevant trunk strength parameter was expressed to body mass (N·m·kg-1) and was used for the statistical analysis. The normalization to the body mass enabled inter-individual comparison (Jaric, 2002). The results of both movement directions in the frontal and transversal plane were averaged for each participant. Additionally, the ratio between trunk flexion and trunk extension, the ratio between trunk lateral flexion right and trunk lateral flexion left, as well as the ratio between trunk transversal rotation right and trunk transversal rotation left, was calculated and used for the statistical analysis.

For the hip and knee strength testing, raw data were recorded at a sampling rate of 200 Hz by the manufacturer's software (IsoMed analyse V.2.0, D&R Ferstl GmbH, Hemau, Germany). All relevant test parameters were computed by a self-developed software written in C++ (Alt et al., 2017b). Each relevant hip and knee strength parameter was expressed to body mass (N·m·kg-1) and the results of both limbs were averaged for each participant (Alt et al., 2017a). For hip strength, the highest isometric peak torque (Nm) for hip abduction and hip adduction was determined. Additionally, the ratio between hip abduction and hip adduction (ABD/ADD) was calculated and used for the statistical analysis. For knee strength testing, the highest peak torque obtained for knee extension and knee flexion in both contraction modes (concentric and eccentric) was determined. Additionally, the conventional knee ratio (knee flexion concentric/knee extension concentric) and the functional knee ratio (knee flexion eccentric/knee extension concentric) were calculated and used for the statistical analysis.

### Statistical Analysis

Data are presented as mean ± standard deviation. In order to discriminate between injured and non-injured players a Decision Tree was optimized using the Classification and Regression Tree (CART) method (Breiman et al., 1984). In each iteration, the method chooses an optimal cut to distinguish between injured and non-injured players. This is done via an exhaustive search that calculates the optimization criteria for every possible cut in all variables. The optimal cut maximizes the reduction of the Gini-impurity from the given observations to the subsequent partition into two nodes starting from all subjects. The criteria is based on the relative frequencies of the two groups in each node as high purity is advantageous but it also involves the relative number of people allocated to each subsequent node because nodes with very few people are not desirable even if they are pure. The process is repeated iteratively for all resulting partitions until the reduction is <0.01 or a partition contains >20 subjects. These termination criteria are used to prevent overfitting to the data. The result is a hierarchical tree-like structure of binary decisions, which is highly interpretable, e.g., *via* a dendrogram.

The association to injury was assessed using a 2 × 2 contingency table based on the Decision Trees classification and the true injury status of the players. Relative risk was calculated as the ratio of the probability of injury occurring in the group, which has been classified by the Decision Tree as injured compared to the probability of injury occurring in the group, which has been classified by the Decision Tree as non-injured (Schmidt and Kohlmann, 2008; Ruddy et al., 2019). Additionally, sensitivity and specificity were calculated from the contingency table according to Ruddy et al. (2019) and serve as performance measures of the Decision Tree. Sensitivity measures the proportion of injured players that were correctly classified by the Decision Tree as being injured, while specificity measures the proportion of non-injured players correctly classified as such (Akobeng, 2007; Ruddy et al., 2019).

## Results

### Epidemiology

Sixty-two elite youth soccer players (age: 17.2 ± 1.1 years; height: 179 ± 8 cm; weight: 70.4 ± 9.2 kg) were enrolled in the study. Twenty-five non-contact injuries were registered over the 2018/2019 season, with 39% of the players (*n* = 24) having one or more non-contact injuries. The overall non-contact injury incidence was 1.2/1,000 h of total exposure time (0.5 injuries per 1,000 h training and 3.9 injuries per 1,000 h competition). The most frequently injured body parts were the ankle (36%) and the thigh muscles (hamstrings 18%, quadriceps 18%), as well as the adductors (16%) and the knee (12%).

### Multivariate Analysis

Table 1 presents the descriptive statistics for all neuromuscular performance parameters obtained from the injury risk screening.

**Table 1:** Descriptive statistics (mean ± standard deviation) of all players investigated according to their injury state (injured/non-injured).

		**Performance parameter**	**All players**	**Injured players**	**Non-injured players**
	Static	COP sway (cm)	119.6 ± 23.7	130.6 ± 25.6	112.4 ± 19.5
Postural control	Dynamic	DPSI	4.54 ± 0.95	4.53 ± 1.04	4.54 ± 0.90
	Unstable	Path of platform (mm)	0.39 ± 0.19	0.45 ± 0.24	0.35 ± 0.15
	Trunk (isometric)	Flex (N·m·kg^−1^)	2.35 ± 0.47	2.26 ± 0.47	2.42 ± 0.46
		Ext (N·m·kg^−1^)	4.98 ± 1.06	4.84 ± 1.00	5.07 ± 1.11
		Flex/Ext (N·m·kg^−1^)	0.49 ± 0.12	0.48 ± 0.14	0.49 ± 0.11
		LatFlex (N·m·kg^−1^)	2.43 ± 0.51	2.29 ± 0.44	2.52 ± 0.54
		LatFlex_r_/LatFlex_l_ (N·m·kg^−1^)	0.98 ± 0.17	0.98 ± 0.12	0.98 ± 0.20
		TransRot (N·m·kg^−1^)	2.00 ± 0.33	1.96 ± 0.34	2.03 ± 0.33
Strength		TransRot_r_/TransRot_l_ (N·m·kg^−1^)	1.02 ± 0.16	1.04 ± 0.18	1.00 ± 0.14
	Hip (isometric)	ABD (N·m·kg^−1^)	1.92 ± 0.31	1.89 ± 0.30	1.94 ± 0.32
		ADD (N·m·kg^−1^)	2.07 ± 0.52	1.98 ± 0.44	2.14 ± 0.57
		ABD/ADD (N·m·kg^−1^)	0.96 ± 0.19	0.98 ± 0.16	0.95 ± 0.20
	Knee (isokinetic)	Qcon (N·m·kg^−1^)	3.09 ± 0.45	2.90 ± 0.46	3.22 ± 0.41
		Hcon (N·m·kg^−1^)	1.68 ± 0.25	1.58 ± 0.19	1.74 ± 0.26
		Qecc (N·m·kg^−1^)	3.65 ± 0.70	3.44 ± 0.68	3.79 ± 0.69
		Hecc (N·m·kg^−1^)	2.14 ± 0.41	2.03 ± 0.34	2.21 ± 0.44
		Conventional Knee Ratio:Hcon/Qcon (N·m·kg^−1^)	0.55 ±0.07	0.55 ± 0.07	0.54 ± 0.07
		Functional Knee Ratio:Hecc/Qcon (N·m·kg^−1^)	0.70 ± 0.12	0.71 ± 0.11	0.69 ± 0.12

*DPSI, Dynamic Postural Stability Index; Flex, trunk flexion; Ext, trunk extension; Flex/Ext, ratio between trunk flexion and trunk extension; LatFlex; trunk lateral flexion; LatFlex_r_, trunk lateral flexion right; LatFlex_l_, trunk lateral flexion left; LatFlex_r_/LatFlex_l_, ratio between trunk lateral flexion right and trunk lateral flexion left; TransRot, trunk transversal rotation; TransRot_r_, trunk transversal rotation right; TransRot_l_, trunk transversal rotation left; TransRot_r_/TransRot_l_, ratio between trunk transversal rotation right and trunk transversal rotation left; ABD, hip abduction; ADD, hip adduction; ABD/ADD, ratio between hip abduction and hip adduction; Qcon, knee extension concentric; Hcon, knee flexion concentric; Qecc, knee extension eccentric; Hecc, knee flexion eccentric; Conventional Knee Ratio, ratio between knee flexion concentric and knee extension concentric; Functional Knee Ratio, ratio between knee flexion eccentric and knee extension concentric*.

**Figure 5:**
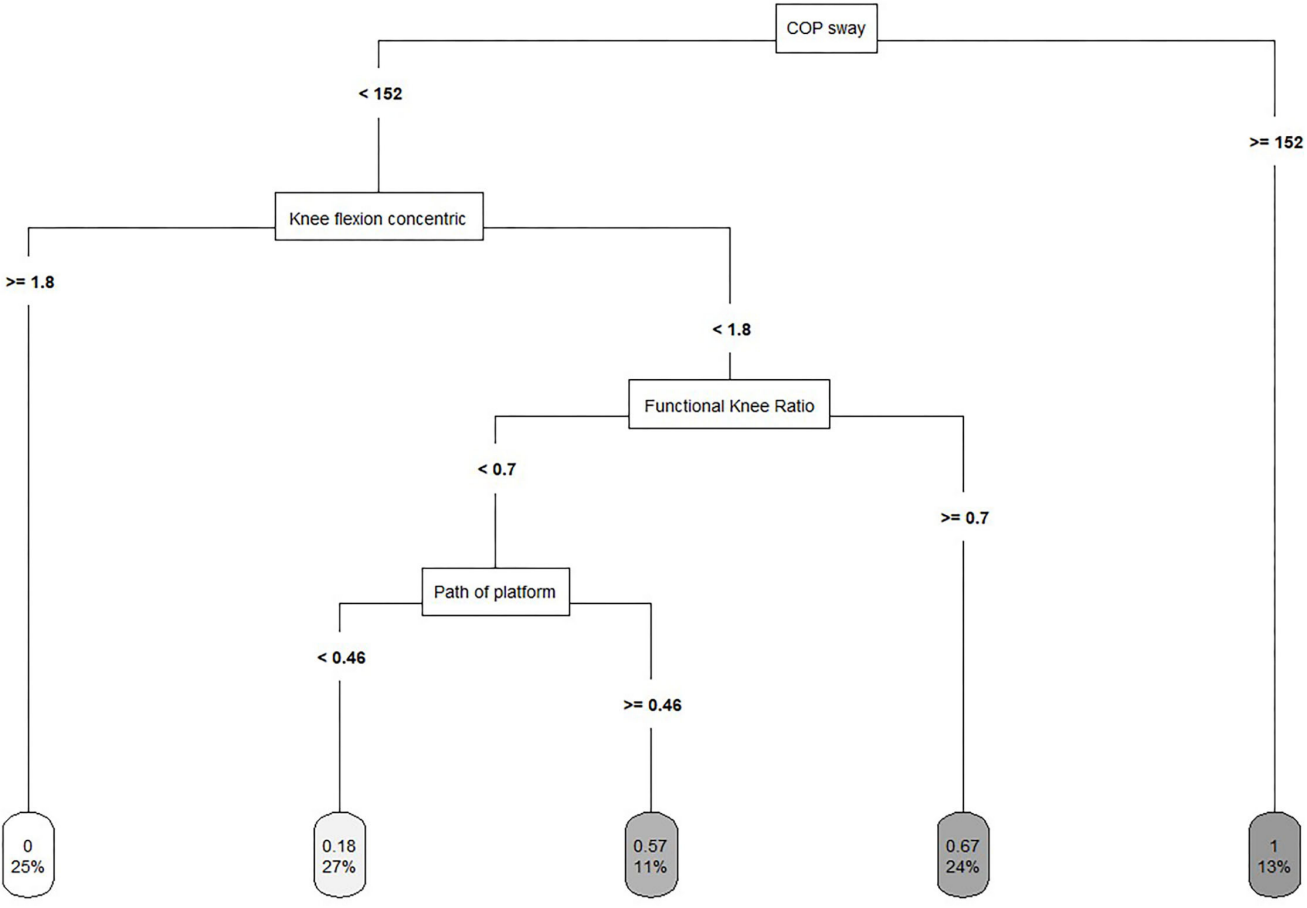
Decision Tree model to discriminate between injured and non-injured players. The lower number in the leaf node indicates the proportion of the sample, whereas the upper number indicates the proportion of the injured players in the node.

The Decision Tree model selected the COP sway, the peak torque for knee flexion concentric, the functional knee ratio and the path of the platform in that hierarchical order as important neuromuscular performance parameters to discriminate between injured and non-injured players (see dendrogram in Figure 5).

The classification of the Decision Tree model showed a sensitivity of 0.73 and a specificity of 0.91. The relative risk was calculated at 4.2, meaning that the risk of suffering an injury is four times greater for a player, who has been classified as injured by the Decision Tree model (see Table 2).

**Table 2:** A 2 × 2 contingency table for the Decision Tree model.

		**Decision Tree classification**	
		**Injured**	**Non-injured**
	**Injured**	22	3
**Injury Status**		True positive (TP)	False positive (FP)
	**Non-injured**	8	30
		False negative (FN)	True negative (TN)

*The frequency distribution of players that have been classified as injured or non-injured by the Decision Tree is displayed against the frequency distribution of players that did or did not suffer an injury*.

## Discussion

Multivariate analysis using a Decision Tree model selected the COP sway, the peak torque for knee flexion concentric the functional knee ratio and the path of the platform as neuromuscular performance parameters associated with the injury outcome. The analysis in this study indicated that players who have been classified as injured by the Decision Tree model had a four-fold increase in their injury risk compared with those who have been classified as non-injured. The properties of the Decision Tree model indicated that this classification correctly identified 73% of injured players as being injured. Furthermore, the classification correctly identified 91% of uninjured players as such. Therefore, measuring static postural control, postural control under unstable condition and the strength of the thigh seem to enable a good indication of injury risk in elite youth soccer players. However, there is an important limitation to interpret the results of the present study due to a small number of injury cases (*n* = 25). Given this limitation, we would like to explicitly point out that the following discussion and the interpretation of the results should be treated with caution. Thus, the present study should be considered as a preliminary study and further studies are necessary to confirm the findings of this study. Nonetheless and to the best of our knowledge, the present study was the first to analyze postural control and the strength of the thigh, trunk and hip muscles as injury risk factors in elite youth soccer players. In this respect, appropriate comparisons with previous studies are limited.

### First Cut of the Optimization of the Decision Tree

Read et al. (2016) reviewed the available pediatric literature for common neuromuscular injury risk factors and proposed a systematic model for youth male soccer players. Dynamic balance has been identified as an injury risk factor. However, the considerations are predominantly based on intervention studies, which have significantly reduced the risk of ankle sprains and enhanced postural stability (McGuine and Keene, 2006). We cannot confirm this finding with our prospective injury risk factor identification data. Nevertheless, postural control seems to be an important neuromuscular performance component in terms of why an injury occurs. In our study, static postural control was selected in the first cut of the optimization of the Decision Tree and served as the root node. The related leaf node includes 13% of the players, of which all sustained an injury. The importance of static postural control measured in a single-legged stance test may be explained by the injured body parts. The ankle was the most frequent body part to sustain an injury (36%). Our findings are in line with Tropp et al. (1984), who found those players showing abnormal stabilometric values to be at an increased risk of ankle injury during the following season compared to players with normal values. McGuine et al. (2000) found that higher postural sway scores corresponded to increased ankle sprain injury rates in high school basketball. Players who demonstrated poor static postural control had nearly a seven-fold increase in ankle sprains compared with those who had good static postural control. However, the technical and physical demands of basketball pose different challenges from those of soccer to the sensorimotor system's ability to protect the neuromuscular system from injury. In contrast to these studies, Frisch et al. (2011) found no significant association between static postural control measured by the excursion of the COP and injury. A plausible explanation for the apparent discrepancy between their and our results could be the testing order. Prior to the testing of static postural control, they asked the players to perform maximal isokinetic tests to assess the muscle strength of the quadriceps and the hamstrings. Muscle fatigue changes the peripheral proprioceptive system by increasing the threshold for muscle-spindle discharge and consequently changing alpha gamma co-activation (Ribeiro et al., 2008). Thus, neuromuscular control, proprioception and functional stability are impaired after intensive exercise, and postural control may decrease (Brito et al., 2012a).

In addition, postural control under unstable conditions was selected in the optimization of the Decision Tree model. It shows that the risk of suffering a non-contact lower extremity injury is higher for players with a higher path of the platform meaning that a lower ability to maintain balance under unstable conditions increase the injury risk. Prospective research on postural control under unstable conditions and the risk of injury is rare. However, research on injury-preventive balance training programs showed that dynamic exercises like single-leg stance on unstable training devices reduce the risk of suffering a lower extremity injury (Caraffa et al., 1996; Myklebust et al., 2003; Olsen et al., 2005). Nevertheless, as postural control under unstable conditions was selected in the fourth cut of the Decision Tree model, the path of the Decision Tree will be discussed later. In summary, according to the systematic model of Read et al. (2016), dynamic balance may be replaced by static postural control and postural control under unstable condition.

### Second Cut of the Optimization of the Decision Tree

Actions in soccer that require rapid decelerations involve substantial eccentric muscle force contributions from the knee extensors, increasing the risk of non-contact ligament injuries (Simonsen et al., 2000). Thus, Read et al. (2016) suggested quadriceps dominance as an intrinsic risk factor and postulated that reduced activation of the hamstrings relative to the quadriceps increases the risk of injury in youth male soccer players. This is partly in line with the results of the present study. Knee flexion concentric was selected as the second splitting variable of the optimization of the Decision Tree. It shows that the risk of suffering a non-contact lower extremity injury is higher for players with lower concentric strength of the hamstring. Additionally, considering the path of the Decision Tree, the related leaf node includes 25% of the players with better static postural control and higher peak torque of knee flexion concentric, of which none suffered an injury. Supporting this finding, lower concentric strength of the hamstrings has been identified as a risk factor in Australian Rules Football (Orchard et al., 1997). In contrast Van Dyk et al. (2016) found no association between lower concentric strength of the hamstrings and the risk of injury within a large cohort study of senior soccer players. In line, the concentric strength of the hamstrings as an injury risk factor was not supported in the meta-analysis by Freckleton and Pizzari (2012). However, Van Dyk et al. (2016) used an inter-subject design comparing injured and non-injured legs and both Freckleton and Pizzari (2012) and Van Dyk et al. (2016) investigated only hamstring strain injuries. These methodological differences make comparisons with the present study difficult.

Although finding small differences in knee extension concentric and knee flexion eccentric between injured and non-injured groups, Van Dyk et al. (2016) concluded that it is not possible to discriminate between injured and non-injured players based on ROC results. This is consistent with the results of our study as neither knee extension concentric nor knee flexion eccentric were selected in the optimization of the Decision Tree although relative differences up to 11% between injured and non-injured players in all movement directions and contraction modes were observed. In contrast, the recent meta-analysis by Freckleton and Pizzari (2012) identified higher concentric strength of the quadriceps as being a risk factor for hamstring strain injuries.

### Third Cut of the Optimization of the Decision Tree

Although the eccentrically co-acting hamstrings have a dynamic role in maintaining the stability of the knee during forceful knee extension (Coombs and Garbutt, 2002), there are conflicting results whether strength imbalances between the hamstrings and the quadriceps (H:Q ratios) are effective in the identification of risk factors in different populations (Soderman et al., 2001; Kim and Hong, 2011; Freckleton and Pizzari, 2012; Van Dyk et al., 2016; Lee et al., 2018).

In the present study, the functional knee ratio was selected as the third splitting variable of the optimization of the Decision Tree. This result is difficult to interpret in that the ratio should potentially be balanced according to normative values to indicate a significant functional capacity of the hamstrings muscles providing muscular stability at the knee joint (Aagaard et al., 1995). Furthermore, the functional knee ratio can be composed in different ways: For example, a higher eccentric strength of the hamstrings and lower concentric strength of the quadriceps indicates a high functional knee ratio weather similar eccentric strength of the hamstrings and concentric strength of the quadriceps indicates as well a higher functional knee ratio. Therefore, the path of the Decision Tree has to be accounted for when interpreting this result.

In principle, the classification shows that the risk of suffering a non-contact lower extremity injury is higher for players with a higher functional knee ratio. The related leaf node includes 24% of the players with better static postural control, lower concentric strength of the hamstring and a higher functional knee ratio, of which 67% suffered an injury. Since this path of the Decision Tree includes only players with lower concentric strength of the hamstrings, we assume that these players also have lower eccentric strength of the hamstrings. Therefore, higher functional knee ratios in this group of players result from both lower eccentric strength of the hamstrings and lower concentric strength of the quadriceps. This leads to the conclusion that players with lower strength of the thigh are at increased risk of injury even if they display better static postural control. However, as there are conflicting results in previous and more recent prospective studies, which also include methodological differences like the choice of the speed of testing, the strength of the knee extensors and knee flexors as injury risk factors must be comprehensively investigated in future studies. Especially instead of using H:Q ratios, which incorporate peak moments emerging at considerable different knee joint angles (Alt et al., 2018), the interpretation of angle-specific strength ratios at extended knee joint angles (e.g., 30°, 20°, and 10° knee flexion) allows to mirror more injury-related knee joint configurations (Ruas et al., 2019). Rather than assessing H:Q ratios at somehow arbitrarily chosen knee flexion angles, the crossover or equilibrium point of eccentric knee flexor and concentric knee extensor moment-angle curves might contribute to a more sensitive identification of players with elevated injury risk (Coombs and Garbutt, 2002; Graham-Smith et al., 2013).

### Fourth Cut of the Optimization of the Decision Tree

Finally, the fourth cut of the Decision Tree model has to be discussed. The related leaf node includes 11% of the players with better static postural control, lower concentric strength of the hamstring, lower functional knee ratio and lower ability to maintain balance under unstable conditions, of which 57% sustained an injury. Although at first it seems surprising that this path of the Decision Tree includes players with better static postural control and lower postural control under unstable conditions, this can be explained based on previous research. In older basic research, postural control has often been treated as a general ability (Ringhof and Stein, 2018). Recent studies have shown that the correlation between static and dynamic (e.g., perturbations of an unstable platform) postural control measures is very low or not given (Granacher and Gollhofer, 2011; Granacher et al., 2011; Fransz et al., 2014; Pau et al., 2015). Therefore, different mechanisms of the postural control system have been suggested to control balance under static and dynamic/unstable conditions (Shimada et al., 2003; Ringhof and Stein, 2018). Furthermore, this leaf node indicates that deficits in the strength of the hamstrings (lower concentric strength in the second cut and lower functional knee ratio in the third cut, which is caused by the assumption that the eccentric strength of the hamstring is lower) may cause an imbalance in muscular co-contraction of the ankle or knee joint (Granacher and Gollhofer, 2011). This can result in reduced joint stiffness during dynamic activities, as indicated by the postural control under unstable conditions in the respective fourth cut of the Decision Tree. Thus, the respective joint is susceptible to injury and the risk of injury is increased in this group of player.

### The Role of the Trunk and Hip Muscles as Injury Risk Factors

Read et al. (2016) propose trunk dominance—predominantly based on a biomechanical view of the trunk—as an injury risk factor. This is in contrast to the results of the present study. Although absolute differences between injured and non-injured players in all anatomic movement planes of the trunk muscles were observed (Table 1), none of the parameters was selected in the optimization of the Decision Tree. Similarly, Leetun et al. (2004) found no relationship between the trunk endurance measures and the injury state in female and male intercollegiate basketball and track athletes. However, despite the widespread acceptance of the importance of trunk muscle endurance and strength, these tests may not accurately reflect trunk muscle function during soccer-specific movements (Huxel Bliven and Anderson, 2013). This is confounded by reports of weak to moderate relationships between the performance on isometric trunk muscle tests and a range of athletic measures (Nesser et al., 2008). Furthermore, we showed in a previous study that core-related Functional Movement Screen items were mainly associated with injury in amateur male soccer players (Kolodziej and Jaitner, 2018). Therefore, the assessment of isolated measures of trunk muscles to infer lower extremity injury risk and performance measures may have questionable validity. Trunk muscle tests that are conducted in a more soccer-specific position and dynamic in nature are required (Leetun et al., 2004).

Strength deficits of the hip muscles are not listed in the systematic model of Read et al. (2016), but they have been examined previously in male soccer players (Engebretsen et al., 2010; Thorborg et al., 2014). Engebretsen et al. (2010) stated that players with weak hip adductor muscles have a four times greater risk of sustaining a groin injury than those without weakened muscles. Partly in line with this, Thorborg et al. (2014) found large eccentric hip adduction strength deficits in players with adductor-related groin pain. These findings cannot be confirmed by the results of the present study. Although absolute differences between injured and non-injured players in the hip muscles were observed (Table 1), none of the parameters were selected in the Decision Tree to distinguish between injured and non-injured players. One potential explanation for the apparent discrepancy between the results could be the method of assessment of hip muscle function. Both Engebretsen et al. (2010) and Thorborg et al. (2014) used a hand-held dynamometer, which has been shown to entail a lack of standardization according to the athletes' starting position, the position of the investigator and the placement of the instrument (Meyer et al., 2013). In contrast, motor-driven dynamometry assessment can be considered highly standardized and reliable for testing abductor and adductor muscle strength (Kolodziej et al., 2019). Nonetheless, the findings are interesting, as this may have consequences for further assessments. Eccentric strength testing of the hip muscles using motor-driven dynamometry may be taken into account, since decreased eccentric muscle strength may impair energy absorption in the tissues, possibly increasing stresses at the muscle tendons and the insertion site (Thorborg et al., 2014).

### Limitations

This study has limitations. Although fatigue has been shown to lead to a higher risk of injury, each injury was weighted equally, regardless of its occurrence during the season. As the physical performance level of the players has a tendency to deteriorate over the season and the overall level of fatigue may accumulate along as the number of matches and training sessions increases, the injury risk has to be evaluated during an etiologically relevant time period – that is, during the time more closely preceding the injury event (Emery et al., 2005; Meeuwisse et al., 2007). Therefore, a single preseason evaluation of players has limited value in predicting injury risk throughout the full season, owing to the changing nature of the risk factor profile, and there is a need for multiple assessments throughout the season. Furthermore, especially in youth soccer players, changes in injury risk at different stages of development seem to be possible (Read et al., 2018). In soccer games with youth male players, injuries also occur more frequently toward the end of the first and second halves. Solely screening players in a non-fatigued state may not accurately identify those individuals whose movement mechanics deteriorate toward the end of a match, affecting their relative risk of injury.

The sample size and injury occurrence in our study are still limited, too. Therefore, caution should be taken into interpretation of the present results. Additionally, the number and distribution of injuries do not allow in-depth analyses by subgroups, such as by types of injuries. Furthermore, one of the methodological approaches of the study was to investigate between-group differences. However, it is important to consider that soccer players are exposed to an asymmetric musculoskeletal loading owing to dominant and non-dominant leg. Therefore, future injury risk analyses of inter-subject differences should be conducted not only between injured and non-injured legs, but also between dominant and non-dominant legs.

Finally, although an injury may occur because of a single risk factor, it is more likely to appear to be the result of an interaction between multiple risk factors. Therefore, we have conducted a multivariate analysis by using a decision tree that maintains the ability of providing distinct cut-off values to validate a screening program in the future and high interpretability. In comparison to multivariate regression analysis, which provides only information about the weighting of the parameters and their interaction, this approach allows drawing conclusions with high practice-relevant implications.

### Practical Considerations

In sports practice, it is desirable to identify all players with an increased risk of injury. The use of the Decision Tree model and the provided cut-off values will allow practitioners and coaches in the future to determine the injury risk of their players after conducting the biomechanical injury risk screening with them. Identifying injury risk factors and assessing individual risk of injury of each player enables customized injury prevention interventions to be provided as part of the player's daily training schedule. Several meta-analyses on the effect of multicomponent exercise prevention programs highlight the role of neuromuscular training and postural control components to be of importance for effective injury risk reduction (Donnell-Fink et al., 2015; Taylor et al., 2015). Therefore, practitioners can build their own customized injury prevention program, which includes strength and postural control exercises of the evaluated multicomponent exercise prevention programs based on the injury risk profile of the player. However, caution should be taken into account when creating injury risk profiles based on the results of the present study as the power of the study is limited due to a small number of injury cases as stated in the Limitation section.

### Conclusion

This research suggested that measuring static postural control, postural control under unstable conditions and the strength of the thigh seem to enable a good indication of injury risk in elite youth soccer players. However, this finding has to be taken with caution due to a small number of injury cases and further studies with larger sample sizes are necessary to confirm the findings of this study. Nonetheless, these preliminary results may have practical implications for future directions in injury risk screening and in planning and developing customized training programs to counteract intrinsic injury risk factors in elite youth soccer players.

## Data Availability Statement

The datasets presented in this article are not readily available because the rights to the data as intellectual property belong to Borussia Dortmund. Requests to access the datasets should be directed to Mathias Kolodziej, mathias.kolodziej@bvb.de.

## Ethics Statement

The studies involving human participants were reviewed and approved by Ethical Committee of the TU Dortmund University. Written informed consent to participate in this study was provided by the participants' legal guardian/next of kin.

## Author Contributions

MK devised the project, designed and performed the experiments, and wrote the paper with input from all authors. MK and KN verified the analytical methods and analyzed the data. MK and MS worked out the technical details of the postural control testing. MK and TA worked out the technical details of the thigh strength testing. TJ supervised the project. All authors contributed to the article and approved the submitted version.

## Conflict of Interest

The authors declare that the research was conducted in the absence of any commercial or financial relationships that could be construed as a potential conflict of interest.

## Publisher's Note

All claims expressed in this article are solely those of the authors and do not necessarily represent those of their affiliated organizations, or those of the publisher, the editors and the reviewers. Any product that may be evaluated in this article, or claim that may be made by its manufacturer, is not guaranteed or endorsed by the publisher.
